# A Hybrid Visual Tracking Algorithm Based on SOM Network and Correlation Filter

**DOI:** 10.3390/s21082864

**Published:** 2021-04-19

**Authors:** Yuanping Zhang, Xiumei Huang, Ming Yang

**Affiliations:** College of Computer & Information Science, Southwest University, Chongqing 400715, China; huangxiumeiswu@163.com (X.H.); yangming@swu.edu.cn (M.Y.)

**Keywords:** visual tracking, deep learning, self-organization mapping network, correlation filter

## Abstract

To meet the challenge of video target tracking, based on a self-organization mapping network (SOM) and correlation filter, a long-term visual tracking algorithm is proposed. Objects in different videos or images often have completely different appearance, therefore, the self-organization mapping neural network with the characteristics of signal processing mechanism of human brain neurons is used to perform adaptive and unsupervised features learning. A reliable method of robust target tracking is proposed, based on multiple adaptive correlation filters with a memory function of target appearance at the same time. Filters in our method have different updating strategies and can carry out long-term tracking cooperatively. The first is the displacement filter, a kernelized correlation filter that combines contextual characteristics to precisely locate and track targets. Secondly, the scale filters are used to predict the changing scale of a target. Finally, the memory filter is used to maintain the appearance of the target in long-term memory and judge whether the target has failed to track. If the tracking fails, the incremental learning detector is used to recover the target tracking in the way of sliding window. Several experiments show that our method can effectively solve the tracking problems such as severe occlusion, target loss and scale change, and is superior to the state-of-the-art methods in the aspects of efficiency, accuracy and robustness.

## 1. Introduction

Object tracking has made remarkable progress in the past two decades [1,2,3], but due to the deformation of the target, sudden movement, light change, severe occlusion, out of field of vision and other factors leading to a large change in appearance, object tracking is still very challenging. In order to cope with these changes, neural networks with memory function and correlation filters are widely used in object tracking. However, the existing tracking algorithms based on the neural network and adaptive model cannot maintain the long-time memory of the target appearance, and the updating of the model in the case of noise may lead to the drifting of the tracking target.

Self-organization mapping neural networks and correlation filters attracted extensive attention in the field of image research and visual tracking [4,5,6]. The popularity of the self-organization mapping neural network (SOM) and associated filters is due to three important properties. First of all, SOM adopts the learning method unsupervisedly, which is more similar to the learning of the biological neural network in the human brain. Its most important characteristic is to self-organize and adaptively change network parameters and structure by automatically looking for internal rules and essential properties in samples [7,8]. Secondly, the correlation filter implements the efficient computation of spatial correlation information through Fourier transformation, thus achieving a higher tracking speed. The correlation filter considers the context information of the target object, and provides more discriminability than the appearance model [9,10] based on the target object only. Even if the target object is severely obscured, the correlation filter can still use context clues to infer the target location. Third, the learning problem of the correlation filter is equivalent to the regression problem [5,6], that is, the cyclically shifted version of the input feature is regressed to soft label data (for example, generated by a narrow bandwidth Gaussian function, with a range of 0 to 1). Therefore, the tracking algorithm based on SOM and correlation filter does not have the fuzziness problem of assigning positive and negative labels to sample data. Based on random sampling of the image area around the estimated target location, the existing detection-based tracking algorithm [4,11,12] trains the tracking classifier in an incremental manner.

Most of the existing tracking methods are based on detection tracking or template matching. The tracking framework based on template matching is represented by Siamese Networks. Siamese Networks originated from a two-steam-based SiamFC [13]. The main advantage of Siamese Networks is that it has found a good balance between tracking speed and tracking performance. However, due to the lack of online updating mechanism, stable tracking effect cannot be achieved in complex scenes, especially in the case of target occlusion, and it is difficult to distinguish the target. The algorithm proposed by Zhong et al. [14,15,16] achieves excellent performance. Taking SiamBAN as an example [15], it solves the disadvantages (length heuristic configuration) caused by the current multi-scale search scheme or predefined anchor box to estimate the scale and aspect ratio of the target. The target tracker based on tracking detection can locate the target location task as a classification problem [17,18,19]. Using the image blocks of target and background, the discriminant classifier is learned online and the decision boundary is obtained. As a representative of the discriminant model, the target tracking algorithm based on correlation filter has made remarkable progress. It is a detection-based tracking algorithm, which uses the target and its surrounding background area to train the classifier online, and the image is transformed from time domain to frequency domain to train the classifier and detect the target. The target tracking algorithm based on a correlation filter can effectively reduce the computational complexity, improve the speed of target detection and meet the real-time requirements of moving target tracking.

This paper proposes a method to dynamically adjust the learning rate of the updated model according to the change of the response peak. When the response peak is high, the tracking effect is better at this time, and multiple models are recorded as candidate models. When the response peak is low, this means the tracking effect is poor and we use the alternative model to update. The linkage estimation of the target scale and the position of the target implement a more efficient target tracking algorithm. A tracking failure detection mechanism and a new detection method are proposed to reduce the impact of model drift caused by tracking failure on the tracking results.

## 2. Related Works

Object tracking has always been an active field in computer vision research, and literature [20,21,22] have made detailed investigations and comprehensive reviews on object tracking. In this section, we will discuss the detection and tracking algorithms that are most closely related to the research algorithms proposed in this paper.

Bolme et al. [18] proposed a tracking algorithm for learning a Minimum Output Sum of Squared Error (MOSE) filter on gray images. The algorithm calculates the correlation between the target image and the correlation filter. The solution of the filter template and the tracking of the target are carried out in the frequency domain. Fast Fourier Transformation (FFT) can be used to achieve fast conversion. Henriques et al. [6] proposed the Circulant Structure of Tracking by Detection with Kernels (CSK) algorithm, which introduces the kernel function into the traditional correlation filter tracking algorithm, the insufficient number of samples in the MOSSE algorithm can leads to a decrease in tracking performance. The circulant matrix is introduced to increase the number of samples while ensuring that the computational complexity does not increase. C. Ma et al. [23] extended the CSK algorithm, using the histogram of gradient (HOG) features, and proposed the Kernelized Correlation Filter (KCF) tracking algorithm ACFLST, and proposed a method to integrate multi-channel features into correlation filtering method which is more robust for extracting the edge information of the object and for lighting and color changes.

**Figure 1:**
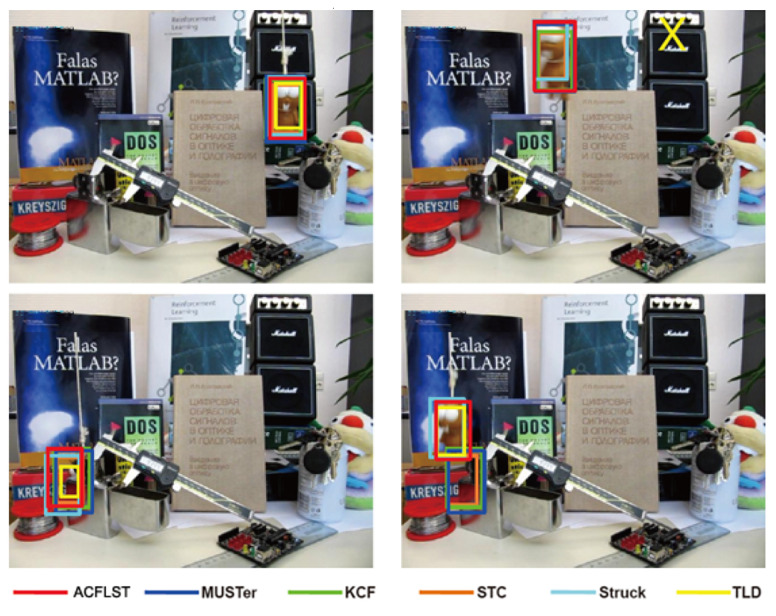
Conclusions of example tracking on the lemming sequence by ACFLST [23], MUSTer [27], KCF [5], STC [24], Struck [4] and TLD [28] (X: no tracking output).

The existing trackers based on correlation filters have achieved certain effects in the field of target tracking, but these algorithms have some defects. These methods use a moving average scheme to update the filter at a high frequency to deal with the time-varying target appearance. This scheme can only maintain the short-term memory of the target appearance. This method may give rise to tracking drift in the presence of noise. Moreover, lack of long-term memory of the appearance of the target is difficult to recover from tracking failure after drifting. As shown in Figure 1, the classic correlation filter tracker (KCF [6], STC [24]) produces target drift due to the noise update of the 4th frame in the video sequence. After 5 frames of severe occlusion, the target tracking failure is caused and unable to recover. These algorithms are limited to predicting the location of the target without predicting the scale of the target, and fail to solve the problem of updating the model when the tracking fails, which limits the performance and application scenarios of the tracking algorithm. The ACFLST [23] algorithm proposed a correlation filter update algorithm, and all the data are relatively excellent in the latest object tracking algorithm test based on correlation filters. However, the performance of the ACFLST algorithm is not ideal. Separated displacement filter and the scale filter are inappropriate in real application scenarios, especially in complicated scenes, because the scale changes of tracking objects are often related to the position. Zhou et al. [25] explored the tracking algorithm of scale-adaptive KCF and deep feature fusion, which improved the feature occlusion problem to a certain extent. Zhang et al. [26] used KCF-based scale estimation to track aerial infrared targets to improve the problem of KCF tracking accuracy decline in the case of large changes in the scale and rotation of aerial infrared targets.

Object tracking adopts detection tracking mode, which treats object tracking as a multiple detection problem in local search window, and usually separates the target from its surrounding background by incremental training classifier, so as to achieve accurate target tracking. Existing methods collect positive and negative training samples from sample areas around the estimated target location and update the classifier with these samples. There may be two problems with this kind of approach. The first problem is sampling uncertainty, i.e., small sample errors may accumulate, and cause the object tracking drift. Many methods have been proposed to reduce the fuzziness of samplings. The main idea of these methods is to intelligently identify and update the classifier when training the characteristics of samples with noise. Examples include Ensemble learning [12,29], Semi-supervised learning [30], Multiple instance learning (MIL) [11] and Transfer learning [31]. The second problem is the stability and adaptability of updating the appearance model. In order to balance the stability and self-adaptability of the algorithm, Kalal et al. [28] decomposed the tracking task into three modules (TLD): tracking, training and detection. The tracking and detection modules can promote each other, provide additional training samples through the results of the tracker and update the detector with effective strategies. The online learning detector can be utilized to reinitialize the tracker in the event of a trace failure, and a similar mechanism is used in [32,33,34] to recover the target object from a trace failure. Zhang [35] et al. used multiple classifiers with different learning rates and designed an entropy metric to fuse multiple tracking outputs. The object tracking algorithm proposed by us uses the online training detector to reinitialize the tracker, this thought is similar to [23,28,35]; however, in our method, only when the memory filter response is under a certain threshold, operation detector is used to detect the drifted objects. This method helps to improve the efficiency of the system while running. Considering the motion continuity of the target, we do not need to apply the detector to target detection in every image frame. In addition, in order to improve the accuracy of the position prediction of the target object, three position filters are adopted.

This paper proposes a long-time tracking algorithm based on multiple correlation filters. Each filter adopts different updating strategies to carry out long-time tracking cooperatively. In this paper, a re-detection mechanism based on support vector machine is designed. Once the target re-enters the field of vision, the algorithm in this paper can recapture the target to track the target. Instead of relying on only one correlation filter [23] for target location estimation, our algorithm is based on SOM network and multiple correlation filters. SOM is used to extract target features, three complementary location correlation filters are used to estimate target location, scale filter is used to predict scale change and memory filter is used to determine the recovery operation in case of tracking failure. The most relevant work with our proposed method is the MUSTer algorithm, which is put forward by Hong [27]. Both methods use the correlation filter based on memory to track. The main differences between MUSTer and our algorithm are the used feature extraction method and the model of target appearance for memory. MUSTer utilizes local feature pool target to represent the appearance of target, our memory filter models the appearance of the target as a whole. It has been shown experimentally that it is often challenging to detect a sufficient number of locally reliable feature points for matching, especially when the target object is of low resolution or unclear structure. Figure 1 shows an example. In the 4th frame, since the detection and matching feature points are very few, the MUSTer tracker cannot recover the object tracking after the tracking fails. At the same time, our proposed algorithm is also relatively close to the algorithm proposed by C. Ma [23], but our proposed algorithm uses three displacement filters, and adopts a joint tracking method in object tracking. This improvement is useful for target positioning and the effect is better.

In this paper, three displacement filters, one scale filter and one memory filter are used to solve the stability and adaptive problems in object tracking. First, we create three displacement filters to estimate the movement of the target. These three filters respectively model the different shapes of the target object and encode the deformation of the target object. In order to accurately locate the target object, we use SOM features to express the basic characteristics of the target. Experimental results show that this feature representation enhances the ability to distinguish between the target and the surrounding background. Secondly, we used the pyramid features to learn scale filter [23], combined with displacement filters, to accurately get the scale of the tracking target. Third, we created a memory filter to track the target. For each tracking result, we calculate the confidence level with the memory filter to judge whether the tracking fails. Once the confidence value is lower than a given threshold, the algorithm starts a SVM detector which is trained online to recover the target.

The essential contribution of our research is to propose a competent object tracking model and algorithm, which effectively uses SOM features, feature pyramid networks and correlation filters to achieve stable and efficient object tracking. Specifically, this method has the following three contributions:

1. Extend our original preliminary work [36] by adding pyramid features and correlation filters, and use an effective target update strategy to update the object detection module [37] to achieve long-time effective tracking of targets.
2. Systematically analyze the influence of different feature types of tracking objects and the size of surrounding environment area on the design of SOM network and correlation filters in complex scenes.
3. The performance of this algorithm and other related works [27] are discussed and compared in detail. We have evaluated the algorithm and conducted extensive testing and comparison on the OTB-50 [38] and OTB-100 [39] datasets and other challenging video sequences (VOT2020 [40], UAV123 [41], LaSOT [42] and NFS [43]).

## 3. Method Overview

**Figure 2:**
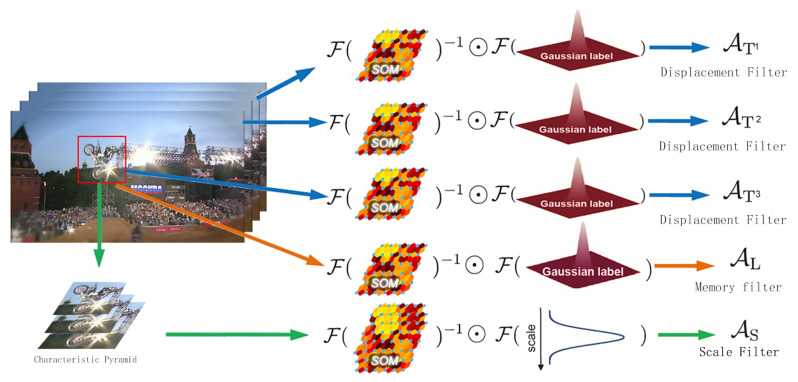
SOM feature extraction and correlation filters. The translation filter $A_{T1},\;A_{T2}$ and $A_{T3}$ with short-time memory adapts to changing appearance of the target and its surrounding context. The long-time memory filter $A_{L}$ is conservatively learned to maintain the long-time memory of the target appearance.

The goal of the object tracking algorithm proposed in this paper is to use SOM and multiple correlation filters to deal with the following challenges in the visual tracking process: (1) the obvious changes in appearance over time; (2) changes in scale; (3) recover the goal from the tracking failure. First, the existing algorithms based on a single correlation filter [23] cannot achieve these goals, because it is tough to strike a balance between stability and adaptability using one filter only. Secondly, although a lot of works have been done to solve the challenge of scale prediction [17,24,44], it is still an unresolved problem because the slight error of scale estimation will cause rapid degradation of the appearance model. Third, it is still a challenge to determine when the tracking failure occurs and to re-detect and track the target from the failure. In the algorithm proposed in this paper, we use three different levels of displacement filters, a scale filter and a memory filter to solve these problems. Figure 2 shows the construction of a correlation filter for visual tracking. The displacement filters $A_{T1},\;A_{T2}$ and $A_{T3}$ are used to model and estimate different forms of targets, respectively, the scale filter $A_{S}$ is used to evaluate the scale estimation of the tracked object, and the long-time memory filter $A_{L}$ is used to keep the long-time memory of the appearance of target to estimate the confidence level of every tracking result.

**Figure 3:**
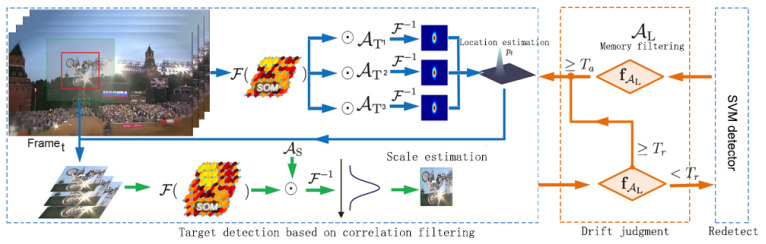
The proposed algorithm diagram.

Figure 3 shows a schematic diagram of the algorithm for object tracking using three correlation filters. It is initialized in the 1st frame of input, and SOM is trained according to the specified object position to extract the regional features, and the three correlation filters proposed by this algorithm are learned. For subsequent input frames, we first use three displacement filters $A_{T1},\;A_{T2}$ and $A_{T3}$ to obtain three target locations at the center of the search window of the previous frame. The average value of these three target locations is our estimated target location. Once the position of estimated target is obtained, we use the scale filter $A_{S}$ to predict the change of the target scale, thereby determining the bounding box of the tracking target. For each tracking result, we judge if the tracking fails (whether the target confidence is lower than a certain set threshold $T_{r}$) by the long-time memory filter $A_{L}$. In the event that the tracker loses a target, the online detector will be activated to recover the lost or drifting target. When the confidence of the re-detected object is greater than the set update threshold $T_{a}$, the long-time memory filter $A_{L}$ needs to be updated first, and then $A_{T1},\;A_{T2}$ and $A_{T3}$ are updated with a reasonable learning rate.

After comparing our experiments with other classifiers, the support vector machine (SVM) can get much better results than other algorithms on the small sample training set. SVM is currently one of the best classifiers with excellent generalization ability and can reduce the requirements for data scale and data distribution. Although the long-time memory filter $A_{L}$ proposed by this algorithm itself can also be used as a detector, because the filter uses high-dimensional features, the calculation load is large. In order to improve the calculation efficiency, we use the online training SVM classifier to construct an additional. We update the detection module and the long-time memory filter $A_{L}$ with a reasonable learning rate, which can snatch the target appearance over a long period of time.

### 3.1. Kernelized Correlation Filters-Based Tracker

Trackers based on correlation filters [17,45] have achieved very good capability in recent evaluations [38,46]. The main idea of these works is regressing the input feature of the cyclic shift to a soft regression index, such as generated by a Gaussian function. The input features of the cyclic shift are similar to the densely sampled samples of the target appearance [6]. Since the training of the correlation filter does not require binary samples (hard threshold), the tracking algorithm using the correlation filter effectively reduces the sampling dilemma that is adversely affected by most tracking algorithms that detect frame by frame. In addition, by using the redundancy in the shifted sample set, Fast Fourier Transform (FFT) can effectively use a large number of training samples to train correlation filters. This increase in training data helps distinguish the target from the surrounding background. This section will explain in detail the derivation process of coring correlation filtering.

Henriques [6] uses cyclic sampling of the target area, that is, dense sampling to reduce the amount of calculation, which not only improves the calculation efficiency, but also improves the tracking accuracy. Different from the sparse sampling methods of other algorithms, the correlation filtering used in proposed method does not strictly distinguish between positive and negative samples, and a transformation matrix is used to cyclically shift the target image block *x*. For a one-dimensional image $x=[x_{1},x_{2},\cdots ,x_{n}]$, the transformation matrix can be as following:

(1) $$P=\left[\begin{array}{ccccc} 0 & 0 & 0 & \cdots & 1\\ 1 & 0 & 0 & \cdots & 0\\ 0 & 1 & 0 & \cdots & 0\\ \vdots & \vdots & \ddots & \ddots & \vdots \\ 0 & 0 & \cdots & 1 & 0 \end{array}\right]$$

The cyclic shift transformation matrix (1) is used to chain-shift the image, and the image transformed by the permutation matrix constitutes the cyclic matrix:

(2) $$X=C\left(x\right)=\left[\begin{array}{c} \begin{array}{cc} x_{1}\;\;\;x_{2}\;\;\;x_{3}\ldots & x_{n}\\ x_{n\;}\;\;x_{1}\;\;\;x_{2}\ldots & \;x_{n-1} \end{array}\\ x_{n-1}\;\;\;x_{n}\;\;\;x_{1}\ldots x_{n-2}\\ \vdots \;\;\;\;\;\;\;\;\vdots \;\;\;\;\vdots \;\;\;\;\;\;\ddots \;\;\;\;\vdots \\ x_{2}\;\;\;x_{3}\;\;\;x_{4}\;\;\;\;\ldots \;\;\;x_{1} \end{array}\right]$$

*X* is the circulant matrix, and the circulant matrix we can use Discrete Fourier Transform (DFT) to obtain the following characteristics:

(3) $$X=Fdiag\left(\hat{x}\right)F^{H}$$

where *F* represents the constant matrix of DFT that transforms the spatial domain data into frequency domain; $\hat{x}=F\left(x\right)$ is the DFT transform of *x* (such as $\hat{x}=F\left(x\right)=F_{x}$), $F^{H}$ is the Hermitian transpose, also called the conjugate transpose matrix, that is, conjugate first and then perform transpose.

*f* is the linear correlation filter which is trained on the image block *X* of size M×N can be regarded as a ridge regression model, which uses all cyclic shifts (horizontal and vertical) of *x* as training data. We assign a regression target score to each shift feature: $y_{i}=\exp\left(-\frac{{\left(m-M/2\right)}^{2}+{\left(n-N/2\right)}^{2}}{2\sigma_{0}^{2}}\right)$, where (m,n) represents the position shifted along the horizontal and vertical directions. In the center of the target object, we have a highest score $y_{i}=1$. If the position (m,n) is far from the target center, the score drops fast from 1 to 0. The kernel width $\sigma_{0}$ is a parameter which is defined previously to control the sensitivity of the scoring function.

First, in the Fourier domain, the ridge regression solution for the circulant matrix *X* is as follows:

(4) $$w\;=\;{\left(X^{H}X+\lambda I\right)}^{-1}X^{H}y.$$

where *I* is the identity matrix with size (M×N)×(M×N), according to Equation (3), we obtain:

(5) $$\begin{array}{cc} X^{H}X & ={\left[Fdiag\left(\hat{x}\right)F^{H}\right]}^{H}Fdiag\left(\hat{x}\right)F^{H}\\ & =Fdiag\left({\hat{x}}^{*}\right)F^{H}Fdiag\left(\hat{x}\right)F^{H}\\ & =Fdiag\left({\hat{x}}^{*}\right)diag\left(\hat{x}\right)F^{H} \end{array}$$

The operations on the diagonal matrix are all element-level, so we get the follows:

(6) $$X^{H}X=Fdiag\left({\hat{x}}^{*}⊙\hat{x}\right)F^{H}$$

Among them, the symbol ⊙ represents the Hadamard product, which is a matrix element-level multiplication, that is, elements with the same position are multiplied separately. Then use the unitarity of the Fourier transform matrix, namely: $FF^{H}=I$, Equation (4) can be rewritten as:

(7) $$\begin{array}{cc} w & ={\left[Fdiag\left({\hat{x}}^{*}⊙\hat{x}\right)F^{H}+\lambda I\right]}^{-1}X^{H}y\\ \; & ={\left[Fdiag\left({\hat{x}}^{*}⊙\hat{x}\right)F^{H}+\lambda FIF^{H}\right]}^{-1}X^{H}y\\ \; & ={\left[Fdiag\left({\hat{x}}^{*}⊙\hat{x}\right)F^{H}+Fdiag\left(\lambda \right)F^{H}\right]}^{-1}X^{H}y\\ \; & ={\left[Fdiag\left({\hat{x}}^{*}⊙\hat{x}+\lambda \right)F^{H}\right]}^{-1}X^{H}y\\ \; & =\left[Fdiag{\left({\hat{x}}^{*}⊙\hat{x}+\lambda \right)}^{-1}F^{H}\right]X^{H}y\\ \; & =\left[Fdiag\left(\frac{1}{{\hat{x}}^{*}⊙\hat{x}+\lambda }\right)F^{H}\right]X^{H}y \end{array}$$

Substituting Equation (3) into Equation (7), we get:

(8) $$\begin{array}{cc} w & =\left[Fdiag\left(\frac{1}{{\hat{x}}^{*}⊙\hat{x}+\lambda }\right)F^{H}\right]{\left[Fdiag\left(\hat{x}\right)F^{H}\right]}^{H}y\\ \; & =\left[Fdiag\left(\frac{1}{{\hat{x}}^{*}⊙\hat{x}+\lambda }\right)F^{H}\right]\left[Fdiag\left({\hat{x}}^{*}\right)F^{H}\right]y\\ \; & =\left[Fdiag\left(\frac{1\cdot {\hat{x}}^{*}}{{\hat{x}}^{*}⊙\hat{x}+\lambda }\right)F^{H}\right]y\\ \; & =Fdiag\left(\frac{{\hat{x}}^{*}}{{\hat{x}}^{*}⊙\hat{x}+\lambda }\right)F^{H}y \end{array}$$

According to the characteristics of the circulant matrix, the construction rule of the circulant matrix and the nature of the Fourier change, we have:

(9) $$C\left(x\right)=Fdiag\left(\hat{x}\right)F^{H}$$

(10) $$C\left(x\right)=C\left(F^{-1}\left(\hat{x}\right)\right)$$

$C\left(x\right)$ is the cyclic shift matrix of x. Synthesizing the right part of the Equations (9) and (10), we have:

(11) $$Fdiag\left(\hat{x}\right)F^{H}=C\left(F^{-1}\left(\hat{x}\right)\right)$$

According to Equation (8):

(12) $$\begin{array}{cc} w & =Fdiag\left(\frac{{\hat{x}}^{*}}{{\hat{x}}^{*}⊙\hat{x}+\lambda }\right)F^{H}y\\ \; & =C\left[F^{-1}\left(\frac{{\hat{x}}^{*}}{{\hat{x}}^{*}⊙\hat{x}+\lambda }\right)\right]y \end{array}$$

According to the nature of the circulant matrix convolution:

(13) $$\begin{array}{cc} F\left(Xy\right) & =F\left[C\left(x\right)y\right]\\ \; & ={\hat{x}}^{*}⊙\hat{y}\\ \; & =F^{*}\left(x\right)⊙F\left(y\right) \end{array}$$

From the Equation (12), we can get:

(14) $$\begin{array}{cc} Fw & =F\left\{C\left[F^{-1}\left(\frac{{\hat{x}}^{*}}{{\hat{x}}^{*}⊙\hat{x}+\lambda }\right)\right]y\right\}\\ \; & =F^{*}\left[F^{-1}\left(\frac{{\hat{x}}^{*}}{{\hat{x}}^{*}⊙\hat{x}+\lambda }\right)\right]⊙F\left(y\right)\\ \; & ={\left(\frac{{\hat{x}}^{*}}{{\hat{x}}^{*}⊙\hat{x}+\lambda }\right)}^{*}⊙F\left(y\right)\\ \; & ={\left(\frac{{\hat{x}}^{*}}{{\hat{x}}^{*}⊙\hat{x}+\lambda }\right)}^{*}⊙\hat{y} \end{array}$$

Since ${\hat{x}}^{*}$ and $\hat{x}$ are in a conjugate relationship, each element in ${\hat{x}}^{*}⊙\hat{x}$ is a real number. Taking the conjugate of such a matrix, the element value does not change in any way. Therefore, Equation (13) can continue to be deduced, as follows:

(15) $$\begin{array}{cc} Fw & =\frac{{\left({\hat{x}}^{*}\right)}^{*}⊙\hat{y}}{{\left({\hat{x}}^{*}⊙\hat{x}+\lambda \right)}^{*}}\\ \; & =\frac{\hat{x}⊙\hat{y}}{{\hat{x}}^{*}⊙\hat{x}+\lambda } \end{array}$$

The following is the objective function of the linear ridge regression training correlation filter:

(16) $$\min_{w}\sum_{i=1}^{M\times N}(f\left(X_{i}\right)-y_{i}{)}^{2}+\lambda {∥w∥}^{2}$$

where λ>0 is a regularization term. Equation (14) is a linear estimator: $f\left(X\right)=W^{T}X$. From Equation (13), the Fourier frequency domain solution is:

(17) $$\hat{w}=\frac{\hat{x}⊙\hat{y}}{{\hat{x}}^{*}⊙\hat{x}+\lambda }$$

where $\hat{x}$ represents the Fourier signal of x, ${\hat{x}}^{*}$ is the complex conjugate transform of x and operation ⊙ is the product of Hadamard. In order to strengthen the discriminative ability of learning filters, Henriques et al. [5] and others introduced the kernel *K*, $K\left(x,x^{\prime }\right)=\varphi^{T}\left(x\right)\varphi \left(x^{\prime }\right)$ which trains the correlation filter in the kernel space, which is used to study the correlation filter in the kernel space when keeping the computational complexity as linear complexity. The calculation formula of the coring correlation filter is:

(18) $$\begin{array}{cc} f\left(z\right) & =w^{T}z\\ \; & ={\left(\sum_{i=1}^{n}\alpha_{i}\varphi \left(x_{i}\right)\right)}^{T}\cdot \varphi \left(z\right)\\ \; & =\sum_{i=1}^{n}\alpha_{i}K\left(z,x_{i}\right) \end{array}$$

where $\alpha =\left\{a_{i}\right\}$ is the dual variable of *W*. In terms of shift-invariant kernels, such as RBF kernels, the dual coefficient α [20,47] can be found by using the cyclic matrix in the Fourier domain:

(19) $$\hat{\alpha }=\frac{\hat{y}}{{\hat{k}}^{{\mathrm{xx}}^{\prime }}+\lambda }$$

where *K* represents the kernel correlation matrix, and the Fourier transform of *K* is as follows:

(20) $$\begin{array}{cc} k^{{\mathrm{xx}}^{\prime }} & =\exp\left(-\frac{1}{\sigma^{2}}{∥x-x^{\prime }∥}^{2}\right)\\ \; & =\exp\left(-\frac{1}{\sigma^{2}}\left({∥x^{\prime }∥}^{2}+{∥x∥}^{2}-2F^{-1}\left({\hat{x}}^{*}⊙x^{\prime }\right)\right)\right) \end{array}$$

Since the algorithm only requires element dot product, FFT and FFT inverse operations, the computational time complexity is O(nlogn), where *n* is the number of input data.

Given a new frame as input, we use the similar solution in Equation (19) to efficiently calculate the correlation response mapping. The method is to crop an image block *z* at the center of the object in the previous frame, and then use the trained target template $\tilde{x}$ to calculate the response map *f* in the Fourier transform domain:

(21) $$f\left(z\right)=F^{-1}\left({\hat{k}}^{\tilde{x}z}⊙\hat{\alpha }\right)$$

Finally, we search for the position of the maximum value of the response map *f* to locate the target.

### 3.2. Displacement Filter

When estimating the target position, we broaden the input bounding box of the target object to include more context around the tracking target and provide more available displacement features. Compared with the tracking algorithm based on the online learning sparse sample classifier [48,49,50] (random sampling surround the estimated target position), our method is based on the correlation filter. The learning sample is intensive, which is all loops of the input characteristics shifted version. The increase in training data helps distinguish the target from the background.

### 3.3. Scale Filter

Danelljan et al. [17] proposed a discriminative correlation filter for scale estimation. We similarly constructed a pyramid feature of the target appearance centered on the estimated position and used it to train the scale-dependent filter. Unlike [17], our method does not use the predicted scale change to update the displacement filter $A_{T}$. Let W×H be the size of the tracking target and *S* be the target scale set. For scale s∈S, the size of the image area captured with the estimated target position as the center is sW×sH, and the captured image block is rescaled to W×H. Then SOM features are extracted from each sampled image block to form a multi-scale representation of the feature pyramid containing the target. Assuming that $X_{s}$ is the feature vector of scale *s*, and $s^{*}$ is the optimal scale of the target object, then:

(22) $$s*=\underset{s}{\arg\max}\left\{\max(f\left(x_{s}\right))|s\in S\right\}$$

In the process of object tracking, our method estimates the change of target displacement firstly, then predicts the change of scale. Our method is different from other existing tracking algorithms, which generally infer changes in position and scale at the same time. For example, the tracking algorithm based on particle filtering [51] uses random samples to approximate the target’s position and scale change state distribution. The gradient descent method (such as Lucas-Kanade [52]) infers the local optimal position and changing scale in an iterative manner. The algorithm we proposed is to break the tracking task into two independent subtasks, which not only reduces the burden of intensive evaluation of the target state, but also avoids the noise update of the displacement filter when the scale estimation is not accurate.

**Figure 4:**
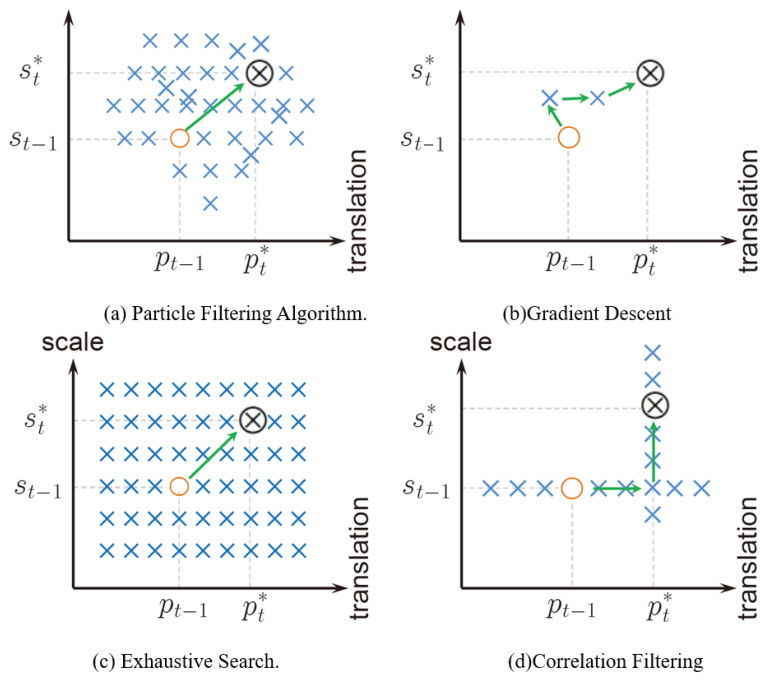
Illustration of common state estimation methods in object tracking. Symbols O, × and ⊗ denote the current, sampled and optimal states, respectively.

The particle filter-based tracking algorithm [51] uses random samples to approximate the target state distribution including position and scale changes, as shown in Figure 4a. Gradient descent methods (such as Lucas-Kanade [52]) iteratively infer local optimal positions and scale changes (see Figure 4b). The object tracking algorithm based on correlation filter [23] decomposes the tracking task into two independent subtasks (position and scale estimation) demonstrated in Figure 4d, which not only reduces the burden of intensive estimation of the target state, but also avoids the noise update of the displacement filter under the circumstance inaccurate scale estimation. Experimental results (see Ablation Study Section). show that the performance of our tracker is significantly better than another implementation (CT-JOP), which uses the estimated scale change to update the displacement filter.

### 3.4. Long-Time Memory Filter

In order to adapt to the changes in the appearance of the target during the tracking process, as time goes by, the tracking algorithm must update the pre-trained displacement filters. However, if the filter is updated by directly minimizing the output error of all tracking results, the computational overhead in the tracking process will be very large [53,54]. The proposed algorithm uses a moving average scheme to update the displacement filter. The updated equation is as follows:

(23) $${\tilde{x}}_{t}=\eta {\tilde{x}}_{t-1}+\left(1-\eta \right)x_{t}$$

(24) $${\tilde{\alpha }}_{t}=\eta {\tilde{\alpha }}_{t-1}+\left(1-\eta \right)\alpha_{t}$$

where *t* is the index of the image frame, and η∈(0,1) is the learning rate. This method updates the position filter every frame, emphasizing the importance of model adaptation and short-time memory of target appearance, but only one of the three position filters is updated each time. The selection of these three filters is a circular selection method. Since this scheme is very effective in dealing with appearance changes, the tracking algorithm [6,17] has achieved good performance in recent benchmark studies [38,46]. However, when the training samples are noisy, these trackers are prone to drift and cannot recover from tracking failures due to the lack of long-time memory of the appearance of the target. The update scheme in Equations (21) and (22) assumes that the tracking result of each frame is sufficiently reliable, so it is natural to use the training sample to update the correlation filter. This is not correct in a complex scene, the result of such an operation is easy to send tracking drift. To solve this problem, we proposed to create a long-time memory filter to preserve the appearance of the target. In order to maintain the stability of object tracking, we set a threshold $T_{a}$ to conditionally update the long-time memory filter. Only when the target’s confidence max(f(x)) is greater than this threshold $T_{a}$ do we update the long-time memory filter. The proposed algorithm uses the maximum value of the correlation response map as the confidence score, because it reflects the similarity between the tracked object and the learning template in the long-time memory correlation filter. Compared with the long-time memory method [55,56] that only uses the first frame as the target appearance, we conditionally update the long-time memory filter to improve its adaptive ability. This allows the long-time memory filter to adapt to a certain degree of time-varying target appearance.

### 3.5. Online Object Detector

The displacement filter $A_{T}$ captures the appearance of the target and is a short-time memory filter. We use contextual information around the target object to learn the filter. In order to reduce the boundary discontinuity caused by the cyclic shift, we weight each channel of the input feature by a two-dimensional cosine window. We use the SOM feature to learn the scale filter $A_{S}$. Unlike the displacement filter $A_{T}$, we directly extract features from the target area without considering the surrounding context, because considering the surrounding context does not provide information about the target scale change. We use a conservative learning rate to learn the long-time memory filter $A_{L}$ to maintain the long-time memory of the appearance of the target to determine whether tracking failure occurs.

Tracking failure is generally caused by some serious occlusion or the target moving out of the camera view. In our tracking algorithm, for each tracked target *z*, we use the memory filter $A_{L}$ to calculate its confidence $\max(f_{A_{L}}\left(z\right))$. Only when the confidence is lower than the predefined re-detection threshold Tr will we activate the detection device. This can reduce the computational load in the object tracking process and avoid using a sliding window for detection in each frame.

In order to ensure the operating efficiency of the system, we use an SVM as a detector instead of using a long-time memory filter $A_{L}$. We intercept training samples at the estimated target position to train the SVM detector incrementally, and assign binary labels to these samples according to their overlap ratio [35]. In this algorithm, we only extract samples with changed targets for training to further reduce the computational workload. During training, the quantized color histogram is used as a feature representation, the image color is converted to the CIE Lab space and each channel is quantized to 5 bits (referring to four equal intervals in each channel). In order to improve the robustness against drastic changes in illumination, we apply the non-parametric local rank transform [57] to the *L* channel.

### 3.6. Method Implementation

As shown in Figure 3, the tracking algorithm proposed in this paper uses SOM features to train three correlation filters ($A_{T1},A_{T2},A_{T3},A_{S},A_{L}$) for position estimation, scale estimation and long-time memory of target appearance. We also built a re-detection module that uses the SVM detector to recover targets from tracking failures. We give a summary of the proposed tracking algorithm in Algorithm 1.
**Algorithm 1:** Object tracking algorithm based on SOM and correlation filter.
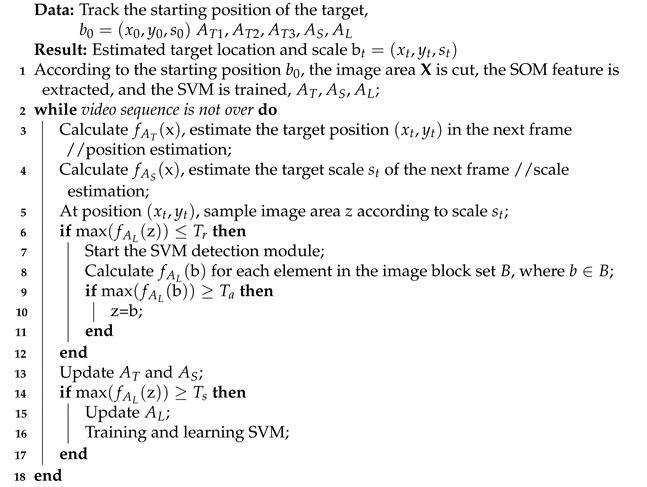


The displacement filter $A_{T1},\;A_{T2}$ and $A_{T3}$ combines the context information to separate the tracking target object from the background. Some methods [20,58] enlarged the target bounding box based on a fixed ratio of 2.5 to include the surrounding context. We conclude through analysis based on experiments that an appropriate increase in the context area will also improve the tracking results. At the beginning, we set it to 2.8 times larger, and then consider the aspect ratio of the target bounding box. We also observed that when the target (such as pedestrian) has a small height and width ratio, the smaller the zoom ratio, the less unnecessary context area in the vertical direction. For this reason, when the aspect ratio of the target is less than 0.5, we reduce the zoom in the vertical direction by half. To train the SVM detector, we densely sample a large window at the center of the estimated target. When the overlap ratio between these samples and the target position is greater than 0.5, we assign them a positive label +1; when their overlap ratio is less than 0.1, we assign them a negative label −1.

In this algorithm, the re-detection threshold $T_{r}$ is set to a lower value of 0.20. When the confidence level $\max(f_{A_{L}}\left(z\right))$ is lower than this value, the algorithm will activate the SVM detection module. When the SVM detection module re-detects the target, the target acceptance threshold Ta is set to 0.4, and only if it is higher than this threshold does it indicate that the target is detected. Each of these detection results needs to be retained during detection, because it is needed when relocating the target and reinitializing the tracking process. We also set the stability threshold to 0.4, and update the memory filter $A_{L}$ when the confidence is greater than this threshold, so as to achieve the purpose of keeping the long-time memory of the target appearance. All thresholds are compared with the confidence score calculated by the long-time memory filter $A_{L}$, and the regularization parameter of Equation (2) is set to $\lambda =10^{-4}$. The Gaussian kernel width setting in Equation (9) is proportional to the target size W×H, $\sigma_{0}=0.15\times \left(W\times H\right)$. The learning rate η=0.01 in Equations (21) and (22). For scale estimation, we use the feature pyramid series N=21, and the scale factor α=1.03.

## 4. Experiments and Results

### 4.1. Experiments Details

We use the latest system of visual tracking evaluation standards to evaluate our methods, including overlap success rate (OS), distance precise rate (DP), OPE, TRE and SRE. Among them, OPE initializes the first frame with the location of the object in the ground-truth, and then runs our tracking method to get the accuracy and success rate.

In the experiment, we followed the experiment rule from the benchmark research [38] and corrected the parameter values of all sequences. The tracking algorithm proposed in this paper is implemented with MATLAB. The computer operating environment is configured as: 32 GB RAM; Intel I7-4770 3.40 GHz CPU.

### 4.2. Experiments on OTB

The dataset here includes OTB-50 [38] with 50 video sequences and OTB-100 [39] with 100 video sequences.

#### 4.2.1. Overall Performance

**Figure 5:**
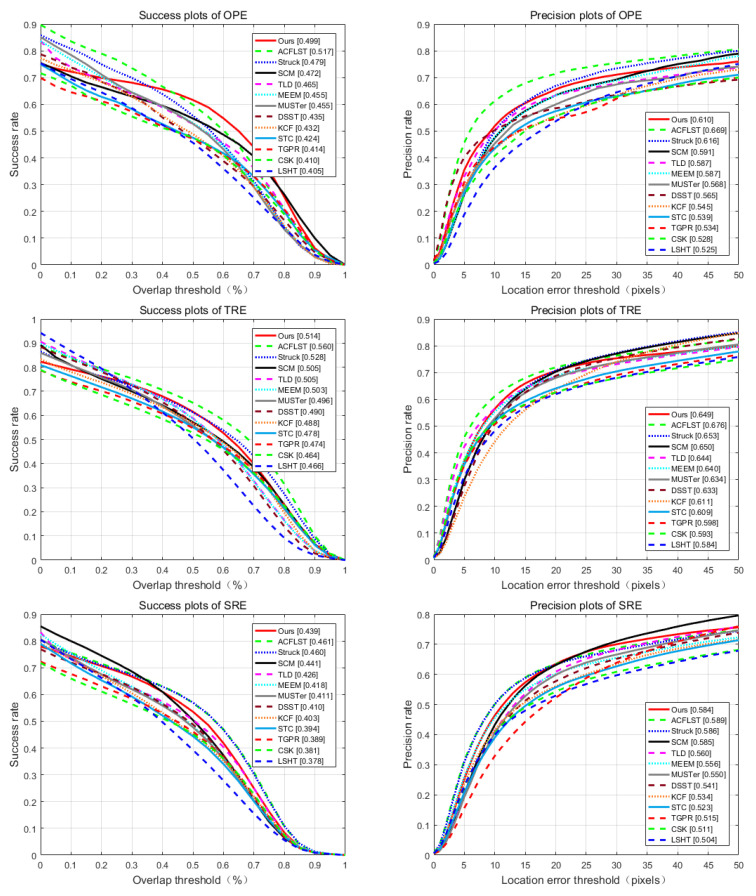
Performance of distance precision and overlap success plots on the test dataset.

In experiments, we initialize overlap success rate at IoU = 0.5, set the distance accuracy rate as 20 pixels. Table 1 shows the OS(Overlap Success), the DP(Distance Precision) and the average tracking speed (the value marked in red is the highest, and the blue is the second highest). Results show that compared with the OTB-50 dataset, the OTB-100 dataset is more challenging because the performance of evaluation from all trackers on OTB-100 is not as good as on OTB-50. In Figure 5, we use one-frame initialization evaluation (OPE—One-Pass Evaluation), temporal robustness evaluation (TRE) and spatial robustness evaluation (SRE) standards to evaluate OTB-100 test sets and the quantitative results were given.

From Table 1, it can be noticed that our algorithm is superior to most current methods in the aspect of overlap success rate and distance accuracy. In terms of overall evaluation results, out algorithm in this paper is second only to SiamBAN [15]. This is mainly because the tracking algorithm in this paper may be sensitive to the initial position given in the first frame, resulting in a slight impact on the accuracy of the initial position. D3S [59] proposed a tracking algorithm using two complementary modules, GIM and GEM, to solve the problem of target dynamic changes. GIM locates the target under high deformation. GEM filters the results and restricts the position of the target when the GIM segmentation target is not unique. Although D3S can restart from tracking failures, it is less effective in dealing with scale changes. Our proposed method has a higher overlap accuracy rate (78.3% vs. 67.6%) than D3S in scale prediction. Both the SiamR-CNN [60] tracker and our proposed method can resolve the scale change of the tracking target, thereby obtaining better overlap accuracy than the D3S tracker. Unlike the SiamR-CNN [60] tracker, we use multiple displacement filters, and update these filters in a cyclic update mode, which can memorize more object appearances and make the tracker more effective in tracking deformed objects. At the same time, we adopted the SOM feature, and updated the displacement filters $A_{T1},\;A_{T2}$ and $A_{T3}$ without considering the scale change. We have observed through experiments that small errors in the scale estimation will cause rapid degradation of the displacement filters $A_{T1},\;A_{T2}$ and $A_{T3}$. In addition, our proposed method has a slightly better overlap success rate than the SiamR-CNN tracker: 78.3% vs. 68.4% on OTB-50, and 69.7% vs. 66.3% on OTB-100.

In terms of tracking speed, our method is at an intermediate level like SiamBAN [15], D3S [59] and PrDiMP [61] trackers. The tracking speed of DiMP [62] and ASRCF [63] is higher than 40FPS. However, these trackers are inferior to our method in terms of accuracy because they cannot recover from failures and cannot handle scale changes. Although it is time-consuming to search and detect using a sliding window when tracking fails, we only activate the detector when the confidence value is lower than the re-detection threshold $T_{r}$, so the speed of the algorithm in this paper is close to the real-time speed of video shooting (20 FPS).

Regarding the TRE and SRE evaluation schemes, the method proposed in this paper cannot get good performance in the OPE evaluation. This is because the TRE and SRE evaluation programs do not fully show the strengths of the methods we propose. The setting of TRE decomposes a video sequence into several segments, so the re-detection importance in long-time tracking is ignored. SRE initializes the tracker with wrong target position and scale. Since our tracker depends on correlation filter training to distinguish the object from the background, inaccurate spatial information of initialization will have a negative impact on the performance of the filter’s target positioning.

#### 4.2.2. Complicated Scenario Test

The test sequence [38] has 11 challenging and complicated scenes, these complex scenes all put forward higher requirements for the object tracking algorithm, such as occlusion or out of view. These complex scenarios are very useful for analyzing the results of the tracker in all aspects. Table 2 and Table 3 show the overlap success rate and distance accuracy test results of the OTB-100 dataset in complex scenarios (the value marked in red is the highest, and the blue is the second highest).

In terms of overlap success rate, the algorithm in this paper is superior to other methods in most attributes (in 11 complex scenarios, the overlap success rate of the algorithm in this paper achieved 6 highest and 3 s highest). Compared with the SiamBAN [15] tracker, our tracker achieves better performance in 5 attributes: illumination variation (0.3%), out-of-plane rotation (4.1%), occlusion (1.4%), deformation (3.1%) and in-plane rotation (4%). In addition, this algorithm is also in the second place in complex scenes with out of view, background clutter and fast motion. We attribute these performance improvements mainly to two advantages. Firstly, we divide the update model of the displacement filter from the model update of the scale filter. Although this method does not seem to be optimal in terms of estimating the target state compared with the SiamR-CNN [60] tracker, it effectively avoids the problem of scale estimation. Degradation of displacement filter caused by the inaccuracy. Secondly, we use the long-time correlation filter as the overall memory template to maintain the appearance of the object. SiamR-CNN [60] uses the information of the first frame and the historical frame for long-term tracking, and iteratively updates the historical frame information. In the presence of obvious deformation and rotation, the key points to identify the target object are much less. In this case, updating the information of the video frame may cause the historical target feature to be blurred, and the tracking performance will decrease. That is why our algorithm has better performance than the tracker of SiamR-CNN in dealing with these challenges.

In terms of distance accuracy, it can be noticed from Table 3 that our method has achieved good results in three aspects: illumination variation (78%), deformation (86.3%) and in-plane rotation (79.5%). These results prove the effectiveness of our algorithm in dealing with large-scale appearance changing in complex scenes and tracking failure recovery. Due to the use of a similar re-detection module, the tracker of SiamBAN and SiamR-CNN also perform very well when processing the situation of fast motion, motion blur and low resolution.

**Figure 6:**
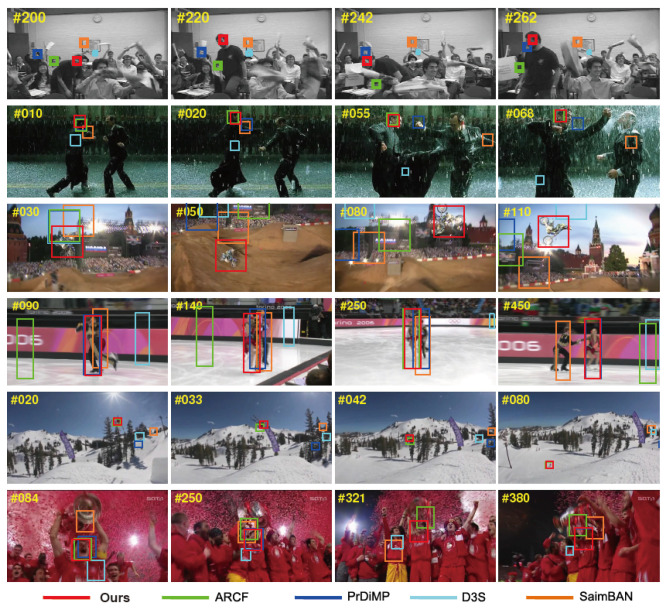
Tacking results on 10 challenging sequences using our algorithm, the ARCF, PriDiMP, D3S and SiamBAN.

We also compared the tracking results of the algorithm proposed in this paper with the four latest object trackers (ARCF [65], PrDiMP [61], D3S [59], SiamBAN [15]). We have selected 6 challenging image sequences for testing, and the test results are shown in Figure 6.

The tracking algorithm we proposed can well calculate the movement and scale change of the object in the challenging image sequence, which can be attributed to three reasons. First of all, our three displacement filter learning is based on the SOM feature that can adaptively unsupervise the appearance of the target, which plays a very important role in obtaining the appearance of the target object. Therefore, the tracker proposed in this paper can achieve a good tracking effect on illumination changing and background clutters, rotation and partial occlusion. Secondly, the update of the scale filter $A_{S}$ and the displacement filter $A_{T}$ is carried out separately, which effectively reduces the degradation of the displacement filter due to the inaccurate scale estimation. Third, in the situation of tracking failure, the online-trained detector can effectively re-detect the target object. For example, in the case of severe occlusion or out of view, the tracker we proposed can restore the target’s tracking.

#### 4.2.3. Ablation Study

In order to well understand the contribution of each part of the tracker proposed in this article, we conducted a component modification study, replacing the SOM network and correlation filter-based objects in this algorithm by using the four-component modified tracker:

1. CT-HOG: Similar to the KCF tracker [6], use HOG features to replace SOM features to train displacement filters $A_{T1},\;A_{T2}$ and $A_{T3}$.
2. CT-NRe (No Re-Detection): Correlation tracker without re-detection module, where the training of displacement filters $A_{T1},\;A_{T2}$ and $A_{T3}$ is based on SOM features.
3. CT-FSC (Fixed Scale): Correlation tracker with re-detection module, but no scale estimation.
4. CT-JOP (Joint Optimization): Similar to DSST [17]] and MUSTer [27] trackers, joint scale change data when updating the displacement filter.

**Figure 7:**
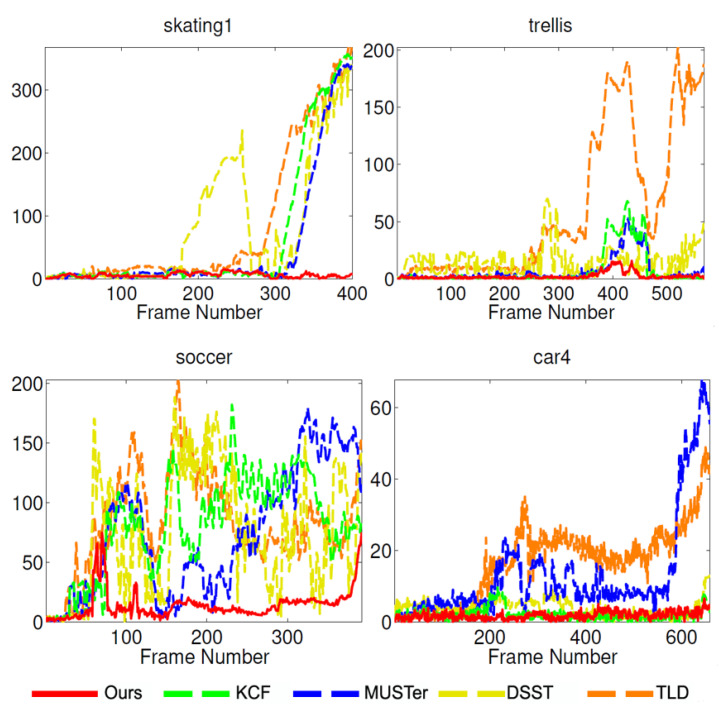
Fames comparison result of central location errors (in pixels) on four challenging sequence.

Figure 6 shows the overlap accuracy and distance accuracy data on the OTB-50 dataset, where IoU = 0.5 and the distance threshold is 20 pixels. Figure 7 lists our comparison of the impact of these algorithms on the tracker in the center position error of each frame of the 4 image sequences. And Table 4 shows component effectiveness analysis on OTB-50 under OPE. Generally speaking, our proposed method can track the objects accurately and stably. Especially on the Soccer sequence, our tracker drifted due to severe occlusion of the target at frame 60, but after a short period of 10 frames, the tracking algorithm we proposed quickly repositioned the target. The result of the effective work of the detection module. Our tracker also drifted out of the field of view in the 400th frame of the trellis image sequence, but it was able to successfully re-detect the target and resume normal tracking in a short time. The performance of CT-NRe method is significantly better than CT-HOG, which illustrates the importance of using SOM features. Comparing CT-FSC and CT-NRe, it can be seen that the use of the re-detection module is very important for the recovery of tracking failure. In addition, the performance of our proposed algorithm with all components is significantly better than the other three implementations (CT-HOG, CT-NRe and CT-FSC). Since we independently updated the displacement filters $A_{T1},\;A_{T2}$ and $A_{T3}$ and the scale filter AS, the distance accuracy of the CT-FSC method is only slightly reduced. The performance of our tracker is significantly better than the CT-JOP method, which shows that the joint update of the displacement filter and the scale filter will lead to a lower tracking effect. It also shows that the scale evaluation is still a challenging problem.

### 4.3. Experiments on VOT2020

We evaluated our algorithm in the short-term tracking dataset and the real-time tracking dataset in the Visual Object Tracking challenge (VOT2020) [40]. Different from the previous VOT, VOT2020 cancels the restart mechanism and replaces it with initialization points. We present the comparison results of the latest trackers submitted to VOT2020 in Table 5. The four best performers are the methods in this paper, RPT [70], OceanPlus [71] and AlphaRef [72] (the value marked in red is the highest, and the blue is the second highest). RPT is a tracker composed of two parts: a target state estimation network and an online classification network [70], whose EAO is the highest in the VOT-ST2020. Its accuracy and robustness are not as good as ours. Our method exceeds RPT by 6.2% and 1.1% on accuracy and robustness in VOT-ST2020. AlphaRef’s [72] performance in VOT-RT2020 is good, and both EAO and accuracy are the best performers, reaching 0.486 and 0.754, respectively. The algorithm in this paper also performed well in VOT-RT2020, both EAO and robustness of ours ranked second, only 0.01 and 0.003 behind the top performer. Our no-reset average overlap also ranked second.

### 4.4. Experiments on NFS

The NFS [43] dataset consists of 100 videos (380*K* frames), which come from real world scenarios using a higher frame rate (above 240FPS) camera. The Area Under Curve (AUC) of each tracker is presented in Table 6, the value marked in red is the highest, and the blue is the second highest and the green one ranks third. Our AUC reached 0.591, which is greater than 0.5, indicating that our tracker has predictive value. Furthermore, our tracker ranks third, and is only 0.003 behind SiamBAN.

### 4.5. Experiments on UAV123 and LaSOT

**Figure 8:**
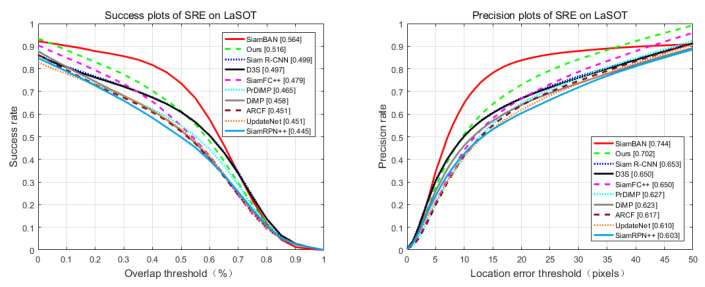
Success and precision plots on LaSOT.

**Figure 9:**
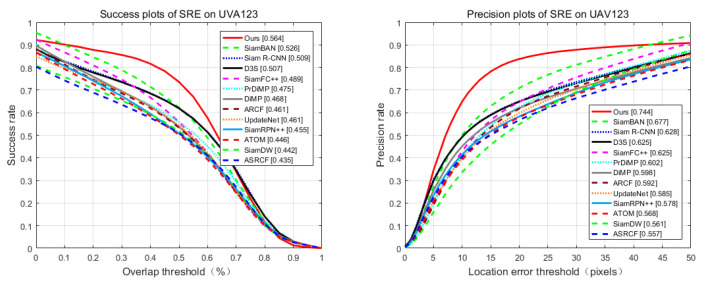
Success and precision plots on UAV123.

We also evaluated our algorithm, SiamBAN [15], SiamR-CNN [60], D3S [59], SiamFC++ [64], PrDiMP [61], DiMP [62], ARCF [65], UpdateNet [66] and SiamRPN++ [67] in the UAV123 [41] and LaSOT [42] datasets. Figure 8 and Figure 9 show the success plots of spatial robustness evaluation (SRE) for each tracker. In LaSOT, the success and precision of our method rank second, and SiamBAN achieves the highest level. However, in UAV123, our method reaches the highest value.

## 5. Conclusions

This algorithm proposes an efficient algorithm for object tracking based on SOM and correlation filters. First of all, three kinds of correlation filters, (1) displacement filter, (2) scale filter and (3) long-time memory filter, are utilized in our algorithm. These three filters work together to obtain the object appearance, object scale and object appearance storage and solve the problem of tracking stability-adaptive problem. The acquisition of target appearance emphasizes the importance of model learning speed and adaptive ability. The long-time memory of the target appearance emphasizes the conservative learning rate and stability of model. The tracking algorithm proposed in this paper takes into account the stability and adaptability of the model in robust tracking. Secondly, in order to improve the positioning accuracy and performance of object tracking, we use SOM features to learn the correlation filters. To improve the tracking results, we studied the influence of the surrounding environment and the learning rate on the tracking efficiency, so as to obtain the optimal scale of the target image area. Third, incremental learning online detector of SVM is introduced to recover the target, and explicitly deal with tracking failures. Experimental results show that the algorithm is better than other state-of-the-art methods in terms of robustness, accuracy and efficiency.

## Figures and Tables

**Table 1:** Total performance on the OTB-50 (I) and OTB-100 (II) datasets.

Tracker	OS (%)		DP (%)		Speed (FPS)	
	I	II	I	II	I	II
Ours	78.3	69.7	84.8	77.2	21.6	22.7
SiamBAN [15]	79.4	70.3	87.5	77.4	23.6	25.2
SiamR-CNN [60]	68.4	66.3	86.5	74.4	10.6	14.2
D3S [59]	67.6	60.2	81	77.1	21.8	23.8
SiamFC++ [64]	60.8	54.5	68.5	65.3	32.5	33
PrDiMP [61]	59.96	55.72	74.01	66.05	23.54	26.99
DiMP [62]	58.08	51.23	75.4	71.28	45.28	45.01
ARCF [65]	34.24	32.37	53.44	52.78	26.82	21.35
UpdateNet [66]	42.15	41.18	54.91	52.76	35.07	29.08
SiamRPN++ [67]	56.79	49.51	65.43	61.26	13.31	11.31
ATOM [68]	39.26	32.1	43.43	41.99	26.29	30.09
SiamDW [69]	50.38	46.26	56.82	61.1	23.17	25.66
ASRCF [63]	50.17	42.05	54.15	51.53	40.79	42.3

**Table 2:** Overlap success scores (%) in terms of individual attributes on the OTB-100 dataset.

Attributes	Ours	Siam BAN [15]	Siam R-CNN [60]	D3S [59]	Siam FC++ [64]	Pr DiMP [61]	DiMP [62]	ARCF [65]	Update Net [66]	Siam RPN++ [67]	ATOM [68]	Siam DW [69]	ASRCF [63]
Illumination variation (23)	73	72.7	72.6	63.3	60.5	59.38	59.38	33.7	41.98	49.85	29.54	44.17	43.16
Out-of-plane rotation (37)	78.8	74.7	71.5	68.5	61	62.91	60.93	40.22	45.23	51.15	37.34	49.99	45.56
Scale variation (28)	69.6	73.3	72.9	57.5	48.2	47.13	52.92	32.39	35.48	48.99	32.71	51.69	33.76
Occlusion (27)	80.2	78.8	74.6	67.9	62	63.69	59.26	34.58	44.12	48.96	36.95	43.3	42.06
Deformation (17)	88.2	85.1	82.5	65.4	67.9	74.36	62.96	34.34	37.88	52.01	42.3	40.59	48.94
Motion blur (12)	66.6	67.7	67.4	67.1	54.5	60.58	51.89	23.78	34.69	52.06	26.71	49.67	27.98
Fast motion (17)	67.1	66.6	65.6	69.7	50	54.94	52.45	24.57	40.01	58.4	35.65	45.67	32.2
In-plane rotation (31)	77.3	73.3	67.9	65.5	58.9	61.48	64.94	36.43	48.74	53.53	34.85	49.01	43.89
Out of view (6)	70.5	71.3	69.7	74.9	55.7	63.37	56.56	28.85	41.92	55.21	38.73	52.16	42.29
Background Clutter (21)	77.7	75.8	77.8	72.3	67.8	66.95	59.75	39.32	47.72	56.41	41.25	41.09	50.19
Low resolution (4)	43.4	46.9	44.7	37.1	31.5	26.91	34.38	33	26.42	24.7	16.54	32.8	19.47
Weighted average	75.7	74.3	72.8	64.8	57.4	59.45	58.26	35.09	41.21	51.32	34.95	45.01	41.12

**Table 3:** Distance precision scores (%) in terms of individual attributes on the OTB-100 datasets.

Attributes	Ours	Siam BAN [15]	Siam R-CNN [60]	D3S [59]	Siam FC++ [64]	Pr DiMP [61]	DiMP [62]	ARCF [65]	Update Net [66]	Siam RPN++ [67]	ATOM [68]	Siam DW [69]	ASRCF [63]
Illumination variation (23)	78	78	79.3	76.7	66.2	70.72	70.61	57.9	47.5	55.62	33.55	49.64	49.04
Out-of-plane rotation (37)	83.1	84.8	85.4	84.1	70	76.86	76.24	56.44	54.76	63.28	50.06	56.56	57.54
Scale variation (28)	76.4	85.2	82.6	79.6	63.2	66.52	74.73	56.3	50.71	65.06	47.24	61.22	50.54
Occlusion (27)	83.7	85.2	84.3	78.5	66.7	77.94	75.93	52.86	53.11	57.46	45.76	54.72	54.31
Deformation (17)	86.3	85.4	85.7	81.3	69.1	82.18	71.63	50	52.35	54.64	50.13	46.8	57.49
Motion blur (12)	65.1	71.1	68.3	72.4	55.6	63.71	59.47	33.19	36.19	54.84	35.98	50.82	35.3
Fast motion (17)	68.1	69.1	69.4	72.7	48.7	58.97	58.39	27.61	38.4	59.73	39.43	55.25	34.78
In-plane rotation (31)	79.5	78.4	79.1	78.7	66.7	72.61	78.52	49.43	54.17	63.18	44.16	60.71	53.8
Out of view (6)	71	71.2	70.5	71.1	50.1	67.28	54.53	41.13	37.17	56.8	39.86	58.83	39.94
Background Clutter (21)	79.8	81.7	83.1	79.3	71.9	77.56	68.21	54.99	60.36	57.89	45.49	40.95	51.96
Low resolution (4)	71	75.9	77.9	88.7	44.9	61.85	72.35	47.37	48.63	55.66	30.98	54.72	57.01
Weighted average	79.4	79.6	80.4	80.5	66.1	72.82	74	50.67	48.2	61.26	44.76	55.6	51.71

**Table 4:** Component effectiveness analysis on OTB-50 under one-pass evaluation (OPE).

	Ours	CT-FSC	CT-NRe	CT-HOG	CT-JOP
OS (%)	79.3	72.9	70.3	60.5	50.7
DP (%)	85.8	83.4	72.5	68.7	61.2

**Table 5:** Results for VOT-ST2020 and VOT-RT2020 challenges. Expected average overlap (EAO), accuracy and robustness are shown. For reference, a no-reset average overlap AO [38] is shown under Unsupervised. (The value marked in red is the highest, and the blue is the second highest).

Tracker	VOT_ST2020			VOT_RT2020			Unsupervised
	EAO	A	R	EAO	A	R	AO
Ours	0.519	0.762	0.87	0.476	0.685	0.821	0.615
RPT [70]	0.530	0.700	0.869	0.29	0.587	0.614	0.632
OceanPlus [71]	0.491	0.685	0.842	0.471	0.679	0.824	0.575
AlphaRef [72]	0.482	0.754	0.777	0.486	0.754	0.788	0.590
AFOD [73]	0.472	0.713	0.795	0.458	0.708	0.780	0.539
LWTL [74]	0.463	0.719	0.798	0.337	0.619	0.72	0.570
D3S [59]	0.439	0.699	0.769	0.416	0.693	0.748	0.508
TRASFUSTm [75]	0.424	0.696	0.745	0.282	0.576	0.616	0.524
AFAT [76]	0.378	0.693	0.678	0.372	0.687	0.676	0.502

**Table 6:** AUC of comparison with state-of-the-art trackers on the NFS dataset in higher frame rate tracking scenarios.

	MDNet [19]	ECO [77]	C-COT [78]	UPDT [79]	ATOM [68]	DiMP [62]	SiamBAN [15]	Ours
AUC↑	0.422	0.466	0.488	0.537	0.584	0.62	0.594	0.591

## Data Availability

The data that support the findings of this study are openly available at http://www.visual-tracking.net, https://cis.temple.edu/lasot/, https://cemse.kaust.edu.sa/ivul/, https://www.votchallenge.net/vot2020/ for dataset OTB100, dataset LaSOT, dataset UAV123, dataset VOT2020 respectively.
